# The roles of adenosine signaling in systemic lupus erythematosus

**DOI:** 10.1016/j.heliyon.2024.e29848

**Published:** 2024-04-17

**Authors:** Ke Dong, Xia-nan Wu, Ying-qi Liu, Lan Yang, Chong Liu, Hui-ping Wang, Zhao-wei Gao

**Affiliations:** a Department of Clinical Diagnose, Tangdu Hospital, Airforce Medical University, Xi'an, Shannxi Province, China; b No. 4 Company, School of Basic Medical Sciences, Air Force Medical University, Xi'an, Shannxi Province, China

**Keywords:** CD39, CD73, Adenosine deaminase, Adenosine, Systemic lupus erythematosus

## Abstract

Systemic lupus erythematosus (SLE) is a complex autoimmune disease with multiple etiological factors. Immune disorder contributes to SLE development and is an important clinical manifestation of SLE patients. Immune dysfunction is characterized by abnormal of B cells, T cells, monocyte-macrophages and dendritic cells (DCs), in both quantity and quality. Adenosine is a critical factor for human immune homeostasis, which acts as an immunosuppressive signal and can prevent the hyperactivity of human immune system. Adenosine levels are significant decreased in serum from SLE patients. Adenosine level is regulated by the CD39, CD73 and adenosine deaminase (ADA). CD39/CD73/ADA catalyzed the cascade enzymatic reaction, which contained the adenosine generation and degradation. Adenosine affects the function of various immune cells via bind to the adenosine receptors, which are expressed on the cell surface. This review aims to export the changes of immune cells and adenosine signal pathway in SLE, as well as the effect of adenosine signal pathway in SLE development.

## 1 Introduction

Systemic lupus erythematosus (SLE) is a multisystem inflammatory autoimmune disease, which is usually affects young and middle-aged women, characterized by production of autoantibodies and autoimmune inflammation. There are multiple factors involved in SLE pathogenesis and development, such as genetics, infection, hormone disorder and immune anomalies [1,2]. Among these factors, immune dysfunction has been found to be the critical driving factor for SLE, which characterized by loss of immune tolerance and overactive immune response. The dysfunction of B cell, T cell, macrophage and dendritic cell (DCs) were all involved in SLE development [[3], [4], [5]].

Adenosine plays as an immunosuppressive signal and shields tissues from an excessive inflammatory response and immune-mediated damage [6]. Zhang et al.’s study has showed that adenosine level was significant decreased in serum from SLE patients [7]. Adenosine metabolism is constituted by enzymes cascade as followed: ATP/ADP were degraded into AMP, which catalyzed by CD39 (ecto-nucleoside triphosphate diphosphohydrolase 1, E-NTPDase1); AMP was degraded into adenosine, which catalyzed by CD73 (ecto-5′-nucleotidase, NT5E); adenosine was degraded into inosine, which catalyzed by ADA. Thus, the dynamic equilibrium of adenosine concentration in vivo were regulated directly by the activities of CD39/CD73/ADA [6]. Human ADA contains two isoenzymes: ADA1 and ADA2. ADA1 is a predomination intracellular enzyme, and play important roles in human immune system development [8,9]. ADA1 gene defects would cause the severe combined immunodeficiency (SCID). ADA2 is a secreted protein produced by monocyte/macrophage [10]. Notably, beside as a adenosine deaminase, ADA2 also belonged to adenosine deaminase growth factor (ADGF) family [11], and could bind to certain immune cell subtype via proteoglycans or other unknown receptor [12]. In our previous study, we have found that ADA activity was significant increased in SLE patients and correlated with the SLE disease activity [13].

There were four types of adenosine receptor (i.e. A1R, A2AR, A2BR and A3R), which were expressed on cytomembrane of various immune cells. Via binding to these adenosine receptors, adenosine regulated a wide variety of cellular processes, including cell development and differentiation, cytokines secretion, cell activity and etc, which behaving as inflammatory response down-regulation and anti-inflammatory immunity up-regulation [14]. Thus, adenosine signal pathway is important for the immune homeostasis and is involved in the pathogenesis of autoimmune diseases.

This study aimed to export the changes of immune cells and adenosine signal pathway in SLE, as well as the effect of adenosine signal pathway in SLE development. In this paper, “adenosine” and “systemic lupus erythematosus” were used as key words to search articles (2002–2022) in PubMed database. Searches were conducted by the authors on Jun 18, 2022. 149 total databases were searched. Correspondence, comment and letters were excluded. Then, manual searches for the reference lists of the literatures was conducted to find potential eligible studies. Finally, 79 papers were included in this study.

Several limitations of this scoping review should be acknowledged. Although we adopted a comprehensive search, relevant studies may have been missed. The present review did not include literature written in languages other than English. This review did not assess risk of bias.

## 2 Adenosine pathway regulated the immune cells of SLE

### 2.1 Adenosine pathway in the regulation of B cells

The production of autoantibodies resulting from the breach of B cell tolerance in SLE patients, which demonstrated the critical role of B cells during the SLE progress. The BAFF (B cell activating factor), also known as Blys (B lymphocyte stimulator), is important in activation, differentiation and survival of B cells. Excess BAFF supports self-reactive B cells and prevent their deletion [15]. Several studies have showed the elevated levels of soluble Blys (sBlys) in serum from SLE patients [16,17]. Belimumab, a recombinant immunoglobulin G1λ human monoclonal antibody targeting Blys, has been approved for the treatment of active SLE [18]. These data demonstrated the important role of B cells abnormality in SLE development.

B cells expressed CD39, CD73 and adenosine receptors. Saze Z showed that 2-chloroadenosine (a adenosine analogue) inhibited B cells proliferation and cytokines expression, and A3R selective antagonist restored B cells functions, which presenting an important role of adenosine signal in B cell biology [19]. Hesse J and colleagues found that the enzymatic activity of CD73 in SLE B cells was almost fully abolished, despite unaltered expression of CD73. while the expression and enzymatic activity of CD39 were not changed [20]. The CD73 inactivation in B cells might contribute to SLE development, because of the less adenosine production. Consistently, CD73 deletion in mice leaded to higher levels of auto-antibodies in response to pristane, and promoted expansion of activated B cells and plasma cells [21]. Dang J and colleagues found that Human gingiva derived MSCs (GMSCs) could alleviate lupus nephritis histopathological scores by suppressing B cells activation, proliferation and differentiation [22]. Notably, the suppressive capacities of GMSCs could be abrogated by CD39^−^CD73 pathway blockage. CD11c^+^ T-bet^+^ B cells were recognized as an important component of humoral immunity and autoimmunity. A2AR agonists could reduce anti-nuclear antibodies and kidney pathology in lupus-prone mice by depleting CD11c + T-bet + B cells [23]. Correspondingly, Bortoluzzi A found that A2AR were up-regulated in lymphocytes from SLE patients (vs healthy subjects). Moreover, there was a negative correlation between the A2AR expression levels and SLE disease activity [24].

### 2.2 T cells

#### 2.2.1 T cells in SLE

**Fig. 1:**
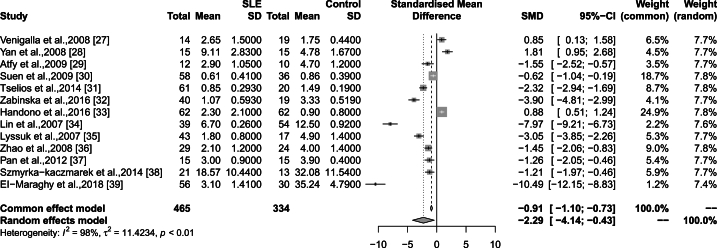
Forest plot showed the Treg cells percentage in SLE patients. 13 studies were used for the forest plot analysis. The total number of healthy control and SLE patients were 334 and 465 respectively.

T cells play important roles in human immune tolerance. Several T cell subsets abnormalities have been described in SLE patients, including regulatory T cells (Treg) and Helper T cell (Th). Treg contributes to immune homeostasis and tolerance. Treg deficits, both quantitative and functional, have been found to be a trigger for SLE development. As shown in Fig. 1, the CD4^+^CD25^+^Foxp3^+^ Treg percentage in CD4^+^ T cells was significant lower in SLE patients compared to healthy controls [[25], [26], [27], [28], [29], [30], [31], [32], [33], [34], [35], [36], [37]]. Moreover, Kamal M's study showed that Treg percentage negatively correlated to SLE disease activity [38]. Valencia X found that Treg from patients with active SLE showed poorly immunosuppressive activity [39]. Notably, Treg/Th17 imbalance have been found to be involved in SLE development. Th17/Treg ratio was positively correlated with SLE disease activity [40]. Via restoring the number of Treg and the balance of Th17/Treg cells, SLE disease activity could be remission [[41], [42], [43]].

#### 2.2.2 Adenosine pathway in regulating of T cells

Accumulated studies have demonstrated the roles of CD39 and CD73 for the immune suppressive activity of Treg cells. Smyth LA's study found that CD73 present on Treg cell derived exosomes was essential for their suppressive function. The suppressive capacity was lacking in the

exosomes derived from a CD73-negative Treg cells [44]. Expanded human Treg in vitro show enhanced suppressive function, which was associated with stable CD73 expression. Co-expression of CD39/CD73 enabled expanded Tregs to convert ATP to adenosine, which is important for its suppressive activity [45]. Moreover, CD39 and CD73 played important roles in regulating T cell differentiation. CD39 and CD73 expression were finely regulated during the transition of naive/memory T cells to effector cells. Naive/memory T cells were endowed with the ability to produce adenosine and lose this ability as they transition to more differentiated cells [46].

ADA also played important roles in T cells immunity, including precursor T cell differentiation, Treg function and Follicular helper T cells (Tfh) function [[47], [48], [49]]. Naval-Macabuhay I's study showed that ADA could lead to a decrease of Treg cells and enhancement of CD4^+^ T cells, which would revert Treg cell-mediated inhibition of immune responses and restore T lymphocyte function in HIV-1 infection [50]. Tardif V et al.’s study have showed that ADA1 expression and activity controled the Tfh program by influencing IL6/IL-2 production [51]. Wu Z's study showed that, although there was no significant alteration of T cell subsets in patients with ADA2 deficiency (DADA2), T cells in DADA2 patients showed distinct cell-cell interactions with monocytes [52].

Adenosine receptors are important for T cell immunity. Ohta A's study showed that Treg cells cultured with A2AR agonist showed increased expression of CTLA-4 and stronger immunosuppressive activity. There was a significant increase of Treg cell number after A2AR stimulation. A2AR-deficiency in Tregs reduces their immunosuppressive efficacy in vivo [53,54]. A2BR was involved in the induction of Tregs. Treg induction could be promoted by A2BR agonist and prevented by A2BR-deficiency [55].

### 2.3 Monocyte/macrophage

#### 2.3.1 Monocyte/macrophage in SLE

Monocyte/macrophages is the important component of the human phagocytic system, which is critical for elimination of pathogens and apoptotic cells. Circulating monocytes can be recruited to tissues and differential into macrophages. Breakdown of immune tolerance in SLE, at least in part, may result from the accumulation of apoptotic cells, which releases a lot of auto-antigens and subsequent induces auto-immune response. Several studies have showed that the phagocytic ability of macrophages to apoptotic cells was decreased in SLE patients [[56], [57], [58]].

The imbalance of M1/M2 polarization of macrophages was closely related to SLE development. The increase of pro-inflammatory M1 cells were found in SLE, and positively correlated to the SLE disease activity [[59], [60], [61], [62]]. Adoptive transplantation M2 cells or inducing of M2 cell proliferation in vivo could alleviate SLE severity in mice models [63,64].

#### 2.3.2 Adenosine pathway in regulating of monocyte/macrophage

Current studies showed that adenosine could promote macrophages polarization toward to M2 and inhibit M1 macrophage activity via binding adenosine receptors [65]. Zavialov AV's study showed that ADA2 could induce monocytes differentiation into macrophages, and stimulates proliferation of T helper cells and macrophages [66]. By using monocyte derived macrophages cells, Tiwari-Heckler S et al.'s study showed that ADA2 treatment could induce up-regulation of pro-inflammatory cytokines: TNFα, IL-1, IL-6 (M1 markers), and downregulation of CD163 (M2 marker) [67]. However, Zhu and colleagues showed that ADA2 promotes M2 polarization of microglia/macrophages [68]. Together, these evidences suggested that the effect of ADA2 on macrophage differentiation may be complex.

CD73 also found to be involved in macrophage polarization process. Xu S et al.'s study showed that CD73 overexpression could promote macrophages/microglia M2 polarization in mice [69]. Consistently, Costales MG et al.'s study showed that CD73 inhibited macrophage produced more NO and a higher levels of pro-inflammatory cytokines (IL-1β, TNF-α), which was a characteristics of M1 macrophages [70].

### 2.4 Adenosine pathway in the regulation of DCs

DCs present phagocytosed antigens to naive T cells and are essential for immunity. Studies have showed that DCs as essential players in the mechanisms underlying SLE. Myeloid DCs (mDCs) and plasmacytoid DCs (pDCs) represent the two major DCs subsets in blood [71]. Chan VS and colleagues have summarized the distinct roles of mDCs and pDCs in SLE [72]. In summary, DCs abnormalities are often observed in SLE patients. Inappropriate activation of DCs leaded to a breach of tolerance and activation of autoreactive T- and B-lymphocytes. The studies have found that unabated activation of DCs through IFN-α produced by pDCs may drive the autoimmune response in SLE [73,74].

Differentiation of functional DCs depends on the microenvironment. The ATP-adenosine balance controls by CD39/CD73 play an important role in DCs regulation [75]. In hypoxic or inflamed tissues, adenosine reached high physiologically levels and regulated the differentiation and immune function of DCs through adenosine receptor. Novitskiy SV et al.’s study showed that adenosine activation skewed DCs differentiation toward a distinct cell population characterized by impaired allostimulatory activity and high expression of angiogenic, pro-inflammatory, immune suppressor, and tolerogenic factors [76]. Liu C et al.’s study showed that stimulation of adenosine-A2AR axis could inhibit the maturation and proinflammatory function of reoxygenated DCs [77]. Moreover, Silva-Vilches C and colleagues found that generation of adenosine by CD73^+^ DCs was a crucial mechanism for the induction of anergic T cells and tolerance [78]. In *vitro*, ADA1 induced mDC maturation and ADA1 treatment also promoted the secretion of the IL-6 from mDCs [79].

## 3 CD39/CD73/ADA1/ADA2 expression levels in immune cells from SLE

**Fig. 2:**
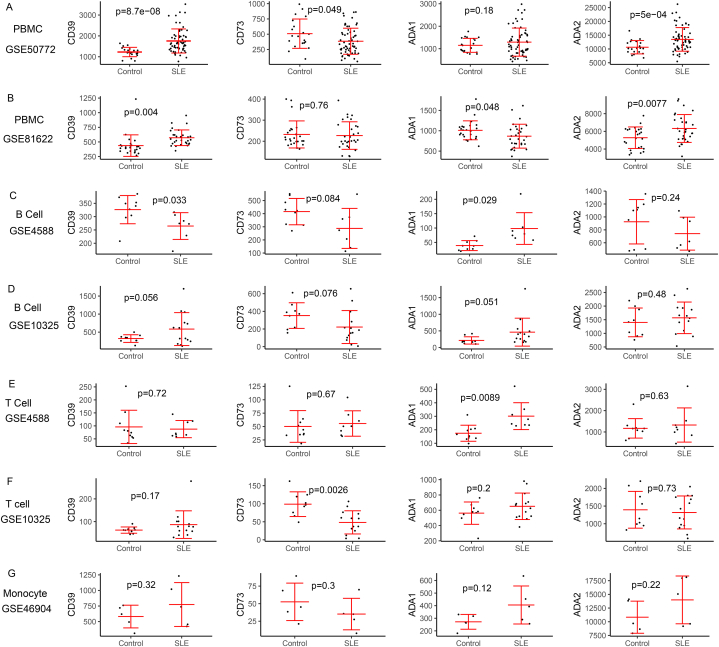
The difference of expression levels for CD39, CD73, ADA1 and ADA2 in SLE and controls base on GEO database. Differential expression analysis of CD39, CD73, ADA1 and ADA2 in PBMC (A, B), B cells (C, D), T cells (E, F) and monocyte (G). Student's t-test was used to analyze the difference expression levels between controls and SLE. *P* < 0.05 was considered to be statistically significant. R software was used to perform statistical analysis and mapping.

Due to the important roles of adenosine metabolism pathway in SLE, we analyzed the expression change of CD39, CD73, ADA1 and ADA2 in SLE peripheral blood mononuclear cell (PBMC) and immune cells subsets, including B cell, T cell and Monocyte based on the GEO database (Table 1), by searching GSE datasets about blood cells from SLE patients in GEO database. As shown in Fig. 2, compared with healthy controls, CD39 and ADA2 expression levels were significant increased in PBMC from SLE patients (Fig. 2A and B). Based on GSE4588 dataset, CD39 was decreased and ADA1 was increased in B cells from SLE, while based on GSE10325 dataset for B cells, there were no significant difference of the expression of CD39, CD73, ADA1 and ADA2 between SLE and healthy controls (Fig. 2C and D). In T cells (SLE vs healthy controls), ADA1 expression was increased base on GSE4588 dataset and CD73 was decreased based on GSE10325 dataset (Fig. 2E and F). In monocyte cells, GSE46904 dataset showed that there were no significant difference of CD39/CD73/ADA1/ADA2 expression between SLE and healthy controls (Fig. 2G). Taken together, these data showed the expression change of molecules related to adenosine metabolism, which indicated that adenosine metabolism might be involved in SLE development.

**Table 1:** Summary of GEO datasets included in this study.

GEO accession	Platforms	Patients	Controls	Cell type
GSE50772	GPL570	61 SLE	20 healthy	PBMC
GSE81622	GPL10558	30 SLE	25 healthy	PBMC
GSE4588	GPL570	7 SLE	9 healthy	CD19^+^ B cell
GSE10325	GPL96	14 SLE	9 healthy	CD19^+^ B cell
GSE4588	GPL570	8 SLE	10 healthy	CD4^+^ T cell
GSE10325	GPL96	14 SLE	9 healthy	CD4^+^ T cell
GSE46907	GPL96	5 SLE	5 healthy	CD14^+^ Monocyte

## 4 Adenosine receptors expression in SLE PBMC and immune cells

**Fig. 3:**
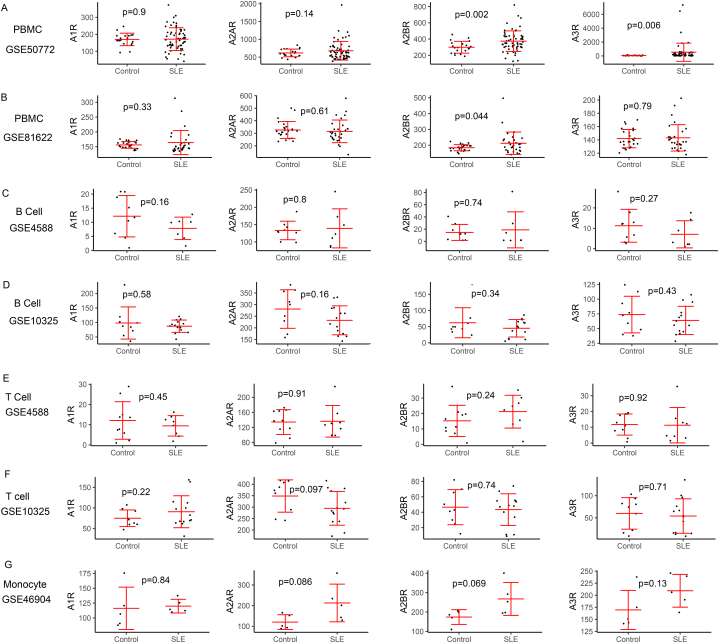
The difference of expression levels for A1R, A2AR, A2BR and A3R in SLE and controls base on GEO database. Differential expression analysis of A1R, A2AR, A2BR and A3R in PBMC (A, B), B cells (C, D), T cells (E, F) and monocyte (G). Student's t-test was used to analyze the difference expression levels between controls and SLE. *P* < 0.05 was considered to be statistically significant. R software was used to perform statistical analysis and mapping.

Adenosine receptors were important for the immune regulated function of adenosine. Thus, we also analyzed the expression change of A1R, A2AR, A2BR and A3R in SLE PBMC and immune cells subsets. As shown in Fig. 3, A2BR expression was significant higher in SLE PBMC than that in healthy controls (Fig. 3A and B). Moreover, GSE50772 dataset showed A3R was significant increased in SLE PBMC (Fig. 3B). However, in B cells, T cells and monocytes, there were no significant difference of four adenosine receptors between SLE and healthy controls (p > 0.05, Fig. 3C–G). Taken together, these data indicated the expression of adenosine receptors might be not extensive changed in SLE patients. Thus, the function of adenosine-receptor signal pathway in SLE might be not regulating by the adenosine receptor expression.

## 5 Concluding remarks

SLE is a complex and systemic autoimmune disease with multiple etiological factors. A better understanding of immunopathogenic mechanisms involved in SLE may be helpful in exploring SLE origin, finding biomarker for diagnose and illness monitoring, and screening the potential treatment targets.

Adenosine is a key signal for human immunity homeostasis, which acts as a “alarm signal” and prevents an overactive immune response. The concentration of adenosine in human is depended by CD39/CD73/ADA1/ADA2, and the effects of adenosine on immune cells are correlated with the adenosine driven-adenosine receptors activation. In this review, we explored the change of immune cells in SLE, and the influence of adenosine signal related molecules on immune cells, including B cells, T cells, monocyte/macrophage and DCs. In summary, the change of quantity or quality for these immune cells are usually involved in SLE development. And the expression or activity levels of CD39/CD73/ADA1/ADA2 may be involved in regulating of these immune cells. Transcriptome sequencing datasets showed that the expression levels of CD39/CD73/ADA1/ADA2 were often changed in immune cells from SLE patients, while the expression of adenosine receptors were not changed (Fig. 2, Fig. 3). To date, the studies on adenosine pathway in SLE are still few, for a better understanding of the immune dysregulation in SLE, more studies involving the adenosine signal pathway in SLE are needed.

## Funding

This work is supported by the Scientist Fund of Tangdu hospital (2021SHRC004; 2021LCYJ049 and 2021SHRC031).

## Ethics declarations

Review and/or approval by an ethics committee was not needed for this study because this study is based exclusively on published literature. For the same reason, informed consent was not required.

## Data availability statement

There is no research related data stored in publicly available repositories, and the data in this paper will be made available on request from the corresponding author.

## CRediT authorship contribution statement

**Ke Dong:** Writing – original draft. **Xia-nan Wu:** Writing – original draft. **Ying-qi Liu:** Writing – review & editing. **Lan Yang:** Data curation. **Chong Liu:** Software. **Hui-ping Wang:** Investigation. **Zhao-wei Gao:** Writing – review & editing, Supervision.

## Declaration of competing interest

The authors declare that they have no known competing financial interests or personal relationships that could have appeared to influence the work reported in this paper.
